# A Toddler With Persistent Crying and a Coin Overlying the Upper Mediastinum

**DOI:** 10.1016/j.acepjo.2026.100443

**Published:** 2026-06-12

**Authors:** Jason Becker, Mark Scheatzle

**Affiliations:** Department of Emergency Medicine, Allegheny General Hospital, Pittsburgh, Pennsylvania, USA

**Keywords:** foreign body ingestion, esophageal ingestion, pediatrics

## 1 Case Presentation

**Figure 1:**
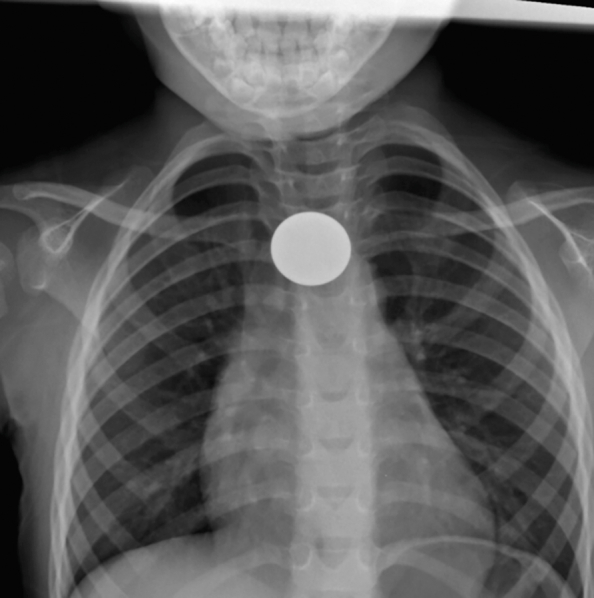
Anteroposterior chest radiograph demonstrating a round radiopaque foreign body overlying the upper mediastinum.

**Figure 2:**
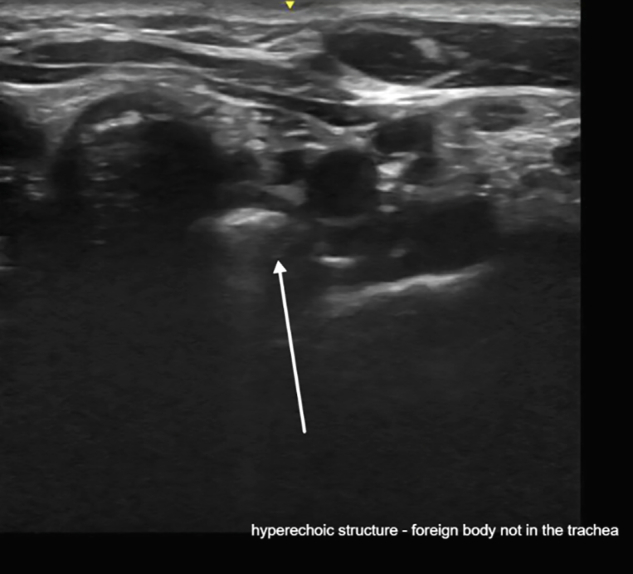
Transverse neck ultrasound demonstrating a hyperechoic foreign body with posterior acoustic shadowing within the esophagus posterior to the trachea (arrow).

**Figure 3:**
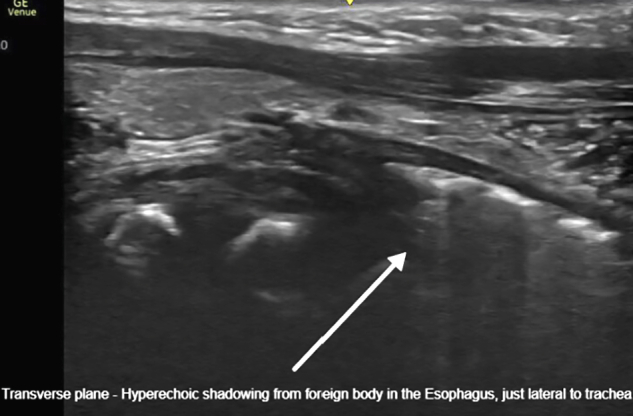
Longitudinal neck ultrasound confirming the foreign body within the upper esophagus (arrow).

A 2-year-old boy presented to the emergency department with 6 h of increased fussiness, persistent crying, and apparent chest discomfort. There was no witnessed foreign body ingestion. He was alert, in no acute distress, and had no stridor, drooling, or increased work of breathing. Anteroposterior chest radiography demonstrated a round radiopaque foreign body overlying the upper mediastinum (Fig 1). Radiographic interpretation raised concern for tracheal versus upper esophageal location. Because transfer to a higher level of care was required for removal, the emergency physician performed a bedside neck ultrasound (Videos 1-3; Figs 2 and 3).

**Video 1:**
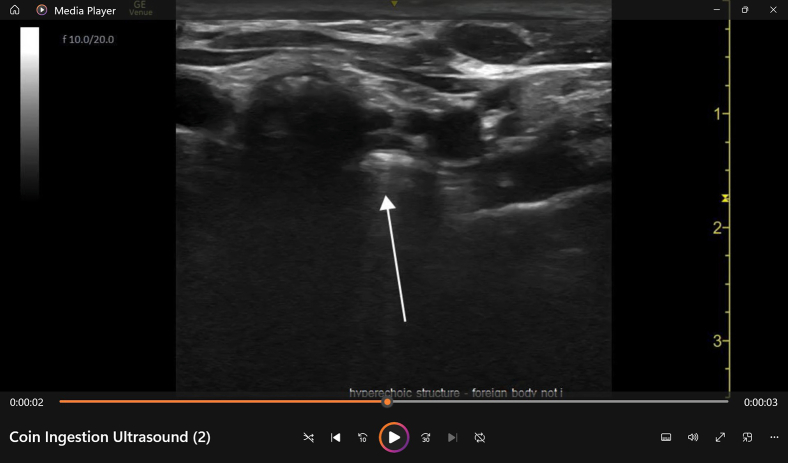
Transverse neck ultrasound demonstrates a hyperechoic foreign body with posterior acoustic shadowing within the esophagus, posterior to the trachea with a labeled arrow.

**Video 2:**
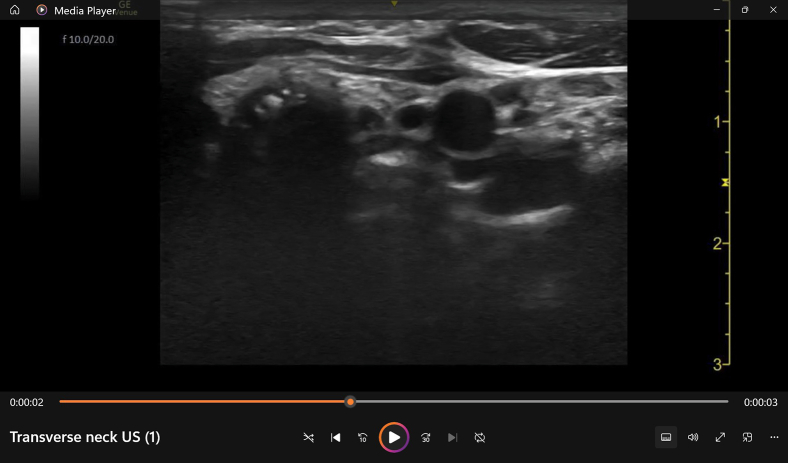
Transverse neck ultrasound demonstrating a hyperechoic foreign body with posterior acoustic shadowing within the distal esophagus, posterior to the trachea.

**Video 3:**
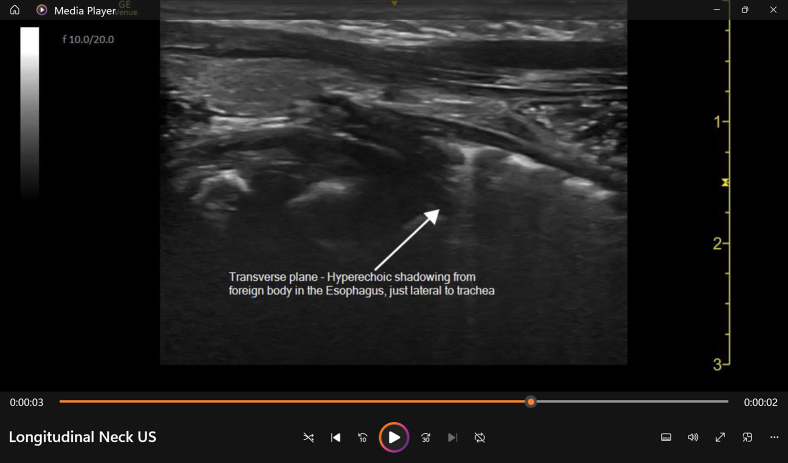
Longitudinal neck ultrasound showing posterior acoustic shadowing within the esophagus, noted by the arrow. The trachea is seen without posterior acoustic shadowing.

## 2 Diagnosis: Upper Esophageal Coin ingestion

Point-of-care ultrasound demonstrated a patent trachea and a hyperechoic foreign body within the upper esophagus, posterior to the tracheal air column (Videos 1-3; Figs. 2 and 3). On transverse neck ultrasound, the trachea appears as a hyperechoic air-mucosa interface with posterior reverberation artifact, whereas the esophagus is typically a collapsed posterolateral structure.^1^ In this clinically stable child with equivocal radiographic localization, bedside ultrasound clarified esophageal rather than tracheal location and guided transfer for endoscopic rather than bronchoscopic management. The coin was subsequently removed endoscopically from the esophagus. Foreign body ingestion is common in young children,^2^ and point-of-care ultrasound may serve as a rapid, radiation-free adjunct when the location of an upper aerodigestive foreign body remains uncertain.^3^^,^^4^

## Funding and Support

By *JACEP* *Open* policy, all authors are required to disclose any and all commercial, financial, and other relationships in any way related to the subject of this article as per ICMJE conflict of interest guidelines (see www.icmje.org). The authors have stated that no such relationships exist.

## Conflict of Interest

All authors have affirmed they have no conflicts of interest to declare.
